# Pain-related analysis on a resorbed ridge with various denture occlusal schemes using finite element method

**DOI:** 10.1590/0103-6440202405798

**Published:** 2024-07-22

**Authors:** David Fatola, Ismet Danial Nasution, Muhammad Sabri, Ricca Chairunnisa

**Affiliations:** 1. Department of Prosthodontics, Faculty of Dentistry, Universitas Sumatera Utara, Medan, Indonesia; 2.Computational & Experimental System Mechanics Research Centre, Department of Mechanical Engineering, Universitas Sumatera Utara, Medan, Indonesia

**Keywords:** bilateral balanced, finite element method, lingualized, monoplane, pressure-pain threshold

## Abstract

Resorbed alveolar ridges, particularly in the lower jaw, have a small denture supporting area, which may cause the stress distribution of mastication load to exceed the pressure-pain threshold (PPT) and induce pain in the mucosa or potentially worsen the ridge resorption. Thus, choosing the ideal occlusal scheme among bilateral balanced (BBO), lingualized (LO), and monoplane (MO) for such conditions becomes crucial. The experiment was conducted using the finite element method on a modeling of a resorbed alveolar ridge in the lower jaw with three dentures placed on top, each of which was given different loading points according to the tooth arrangement of BBO, LO, and MO. The axial load was 100 N, and the resultant oblique loads on BBO and LO were 119 N and 106 N, respectively. The von Mises stresses for BBO, LO, and MO were observed in nine denture-supporting areas, and the results showed that the axial load did not produce stresses that exceeded the PPT value (0.64925 MPa) for BBO, LO, and MO with the highest value on area H, 0.43229 MPa, 0.39715 MPa, and 0.31576 MPa, respectively. However, the oblique load direction showed that the BBO had more areas (area E 0.80778 MPa and area H 0.76256 MPa) that exceeded the PPT than LO (area E 0.64394 MPa). The lingualized occlusal scheme is ideal for patients with resorbed alveolar ridge conditions, especially in terms of limiting interferences when the denture is functioning while maintaining comfort but still providing good masticatory performance and satisfactory esthetics.

## Resumo

Os rebordos alveolares reabsorvidos, especialmente na mandíbula inferior, têm uma pequena área de suporte para prótese, o que pode fazer com que a distribuição de estresse da carga mastigatória exceda o limiar de pressão-dor (PPT) e induza dor na mucosa ou piore potencialmente a reabsorção do rebordo. Assim, a escolha do esquema oclusal ideal entre balanceado bilateral (BBO), lingualizado (LO) e monoplano (MO) para essas condições torna-se crucial. O experimento foi conduzido usando o método de elementos finitos em um modelo de um rebordo alveolar reabsorvido na mandíbula inferior com três dentaduras colocadas na parte superior, cada uma das quais recebeu diferentes pontos de carga de acordo com o arranjo dentário de BBO, LO e MO. A carga axial foi de 100 N, e as cargas oblíquas resultantes em BBO e LO foram de 119 N e 106 N, respectivamente. As tensões de von Mises para BBO, LO e MO foram observadas em nove áreas de suporte da dentadura, e os resultados mostraram que a carga axial não produziu tensões que excedessem o valor PPT (0,64925 MPa) para BBO, LO e MO com o valor mais alto na área H, 0,43229 MPa, 0,39715 MPa e 0,31576 MPa, respectivamente. Entretanto, a direção da carga oblíqua mostrou que o BBO tinha mais áreas (área E 0,80778 MPa e área H 0,76256 MPa) que excediam a PPT do que o LO (área E 0,64394 MPa). O esquema oclusal lingualizado é ideal para pacientes com condições de rebordo alveolar reabsorvido, especialmente em termos de limitação de interferências quando a dentadura está funcionando, mantendo o conforto, mas ainda proporcionando um bom desempenho mastigatório e uma estética satisfatória.

## Introduction

Edentulism is viewed as an irreversible condition and the end consequence of oral health diseases, which result in physical alterations and health conditions such as biomechanical, phonetic, and aesthetic appearance abnormalities that lower quality of life ^1^. A recent systematic review and meta-analysis found that the global prevalence of edentulous individuals is 22% ^2^. Socioeconomic factors, educational background, and government policies play roles for most patients, believing that tooth extraction and denture fabrication are more financially advantageous treatment alternatives than treating more complex dental issues and incurring larger costs ^3^.

Edentulous patients have a smaller denture supporting area due to ongoing resorption, as in the case of resorbed ridges ^4^. Numerous studies also revealed a negative relationship between age and alveolar ridge height ^3^ ^,^ ^5^, this is thought to be due to the rate of bone resorption, which cannot be balanced by the rate of bone formation ^6^. Recent studies even indicated that an imbalance in stress distribution under the denture might accelerate ridge resorption ^6^ ^,^ ^7^.

If excessive pressure is applied to the oral mucosa, it has a mechanical and physiological capability that can lead to pain or discomfort and even more bone resorption ^8^ ^,^ ^9^. The maximum capability is described as the pressure-pain threshold (PPT), in which the pain occurs when the ridges receive excessive pressure beyond its limit ^8^. Physiologically, the mandibular ridge gets its blood supply from the periosteal plexus vessels, which are susceptible to disruption when exposed to pressure, thus triggering pain and discomfort. If pressure continues, inflammatory cells will become involved, causing hydrostatic pressure that exceeds capillary pressure. As a result, the supply of nutrients will be hampered, resulting in progressive resorption, which causes a resorbed ridge condition ^10^.

Efforts that can be made to avoid pain include minimizing the load that will be received by the ridge by avoiding foods that require large chewing forces or by reducing resistance when the denture is functioning by selecting a certain occlusal scheme ^11^. The bilateral balanced occlusal scheme with the use of anatomical denture teeth results in more interferences during the mastication process, causing uneven load distribution on the alveolar ridge and resulting in pain ^12^. Therefore, the choice of other occlusal schemes such as lingualized or monoplane, is more widely used in resorbed ridge conditions, although there are concerns that these occlusal schemes may also affect aesthetics and masticatory performance ^11^.

A complex simulation of the stomatognathic system is necessary to be able to examine how the biomechanical behavior happens because the evaluation of the mucosal response under the denture when it is functioning results in a complex structural response ^13^. The rapid development of digital technology allows for alternative testing other than in vivo and in vitro tests, simulation modeling can be carried out accurately, and complex biomechanical behavior can be simulated repeatedly without any destructive processes, in the form of in silico testing, in the form of finite element methods (FEM) ^13^ ^,^ ^14^. This study aims to analyze how the masticatory load is distributed under dentures with bilateral balanced, lingualized, and monoplane occlusal schemes in the condition of the resorbed ridge of the lower jaw using the finite element method and its relationship with pain.

## Material and methods

The lower jaw resorbed ridge model was obtained from the impression of a patient with class III ridge height, according to the American College of Prosthodontics (ACP). Three different occlusal schemes - bilateral balanced (BBO), lingualized (LO), and monoplane (MO) - were used to set up complete dentures. The denture was prepared with heat-polymerized acrylic resin (BasicQ Vertex® shade 20; Vertex-Dental B.V.; Netherland), and occlusal adjustment was carried out during the remounting process. Three mandibular dentures and one mandibular resorbed ridge model were then scanned using an intraoral scanner (iTero ElementTM Plus Series; Align Technology Inc; Arizona, USA). The data in STL format was then reconstructed using a 3D modeling application (SpaceClaim 2020, USA) and exported into a finite element method application (ANSYS Parametric Design Language; ANSYS Inc; USA).

The modeling condition was in the form of close contact between the denture and the ridged mucosa but not fused, thus allowing movement of the denture with a friction coefficient of 0.16, which was assumed to be the friction coefficient due to the presence of saliva ^15^. Numerically, the anatomical side of the denture was referred to as the contact surface, and the mucosal surface of the ridge was referred to as the target surface. This resorbed ridge modeling of the lower jaw was given fixed support underneath as an assumption that the mucosa was attached to the alveolar bone.

The resorbed ridge modeling was assumed to be linear isotropic with an average Young's modulus of the ridged mucosa of 3.5 MPa ^15^ and a Poisson ratio of 0.49 ^15^ with a thickness of 1.49 mm ^16^ ^,^ ^17^. Modeling of three mandibular dentures made of heat-polymerized acrylic resin with bilateral balanced, lingualized, and monoplane occlusal schemes with an average Young's modulus of 1,960 MPa ^18^ and a Poisson's ratio of 0.3 ^18^. Next, 3D geometric reconstruction modeling was created using mesh automatically in Ansys with a triangular shape totaling 373,398 - 418,831 elements and 595,866 - 688,843 nodes.

**Figure 1:**
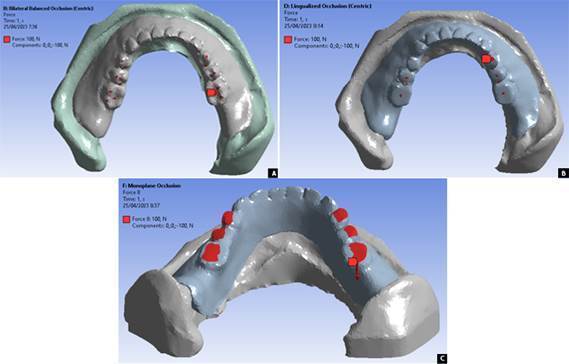
Masticatory load points in the axial direction for denture occlusal schemes of bilateral balanced (A), lingualized (B), and monoplane (C)

**Figure 2:**
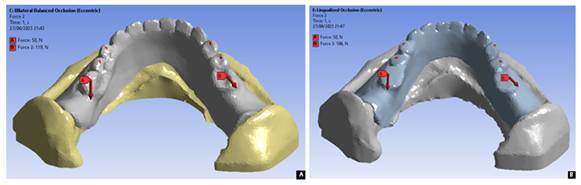
Masticatory load points in an oblique direction for denture occlusal schemes of bilateral balanced (A) and lingualized (B)

The masticatory load was applied according to the force conditions in the oral cavity. A load in the axial direction of 100 N ^15^ ^,^ ^19^ was applied to the occlusal surfaces of the premolars and molars on the right and left sides to represent the axial direction of the masticatory load and the load in the oblique direction with a resultant load of 119 N for the BBO occlusal scheme (the calculation based on the occlusal slope of anatomical denture teeth 33⁰) ^18^ ^,^ ^20^ and 106 N for the LO occlusal scheme (the calculation based on the occlusal slope of semi-anatomical denture teeth 20⁰) ^18^ ^,^ ^20^ to represent the direction of the masticatory load laterally with a balancing side load of 50 N ^19^ ^,^ ^21^, which was adjusted to the load point pattern in each occlusal scheme in a balanced manner as seen in Figure 1 and Figure 2.

The stress distribution value generated in the ridged mucosa under the denture was expressed in MPa and analyzed in the form of a von Mises stress color contour plot ^15^ which was then compared with the PPT value of each area of the denture supporting mucosa, which is divided into nine areas as shown in Table 1.

**Table 1:** Denture supporting mucosa in the mandible and the PPT value ^17^

Area	Denture Supporting Areas in the Mandible	PPT Value (MPa)
A	The buccal surface of the anterior ridge	0.30625
B	Alveolar crest of the anterior	0.35525
C	Alveolar crest of the canine eminence, posterior lingual vestibule, lingual surface of the molar region	0.40425
D	Alveolar crest of first premolar area, buccal and lingual surface of the premolar region, buccal vestibule of the molar region	0.45325
E	Alveolar crest of second premolar area	0.50225
F	The buccal surface of the first molar region, anterior buccal shelf	0.55125
G	Buccal shelf	0.60025
H	Alveolar crest of the first molar region, the buccal surface of the second molar region	0.64925
I	Alveolar crest of second and third molars, retromolar pad	0.69825

## Results

The FEM simulation test was run to see the stress distribution generated in the axial load direction for the BBO, LO, and MO occlusal schemes while in the oblique load direction for the BBO and LO occlusal schemes.

**Figure 3:**
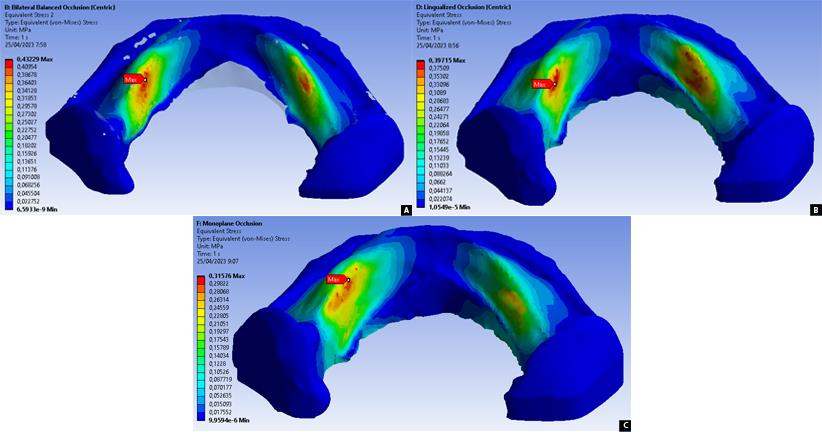
Stress distribution resulted from axial masticatory load for denture occlusal schemes of bilateral balanced (A), lingualized (B), and monoplane (C)

**Figure 4:**
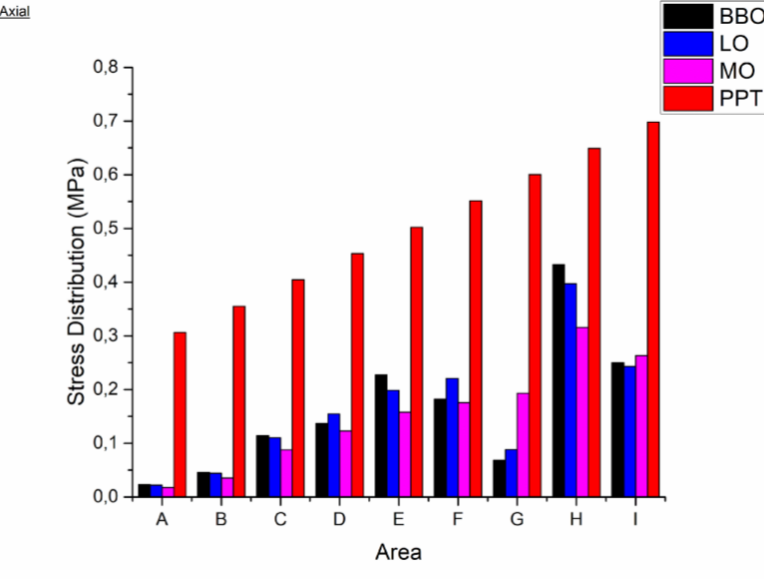
Graph of stress distribution on resorbed alveolar ridge under denture occlusal schemes of bilateral balanced, lingualized, and monoplane when received masticatory load in axial direction compared to PPT

In the direction of an axial load of 100 N for the BBO, LO, and MO occlusal schemes, the resulting stress distribution showed the highest stress concentration in the H area of ​​0.43229 MPa, 0.39715 MPa, and 0.31576 MPa, as seen in Figure 3 and Figure 4. Overall, no stress distribution exceeded the PPT value in the three occlusal schemes when receiving loads in the axial direction. The BBO occlusal scheme showed the highest stress distribution in areas A, B, C, E, and H compared to the other two occlusal schemes. The LO occlusal scheme showed the highest stress distribution in areas D and F compared to the other two occlusal schemes. The MO occlusal scheme showed the highest stress distribution in areas G and I compared to the other two occlusal schemes.

**Figure 5:**
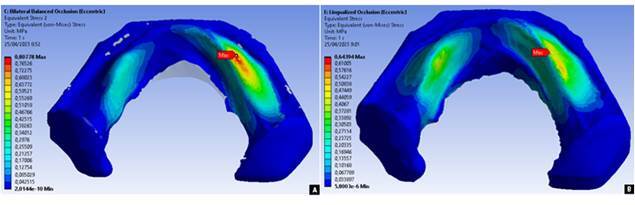
Stress distribution resulted from oblique masticatory load for denture occlusal schemes of bilateral balanced (A) and lingualized (B)

**Figure 6:**
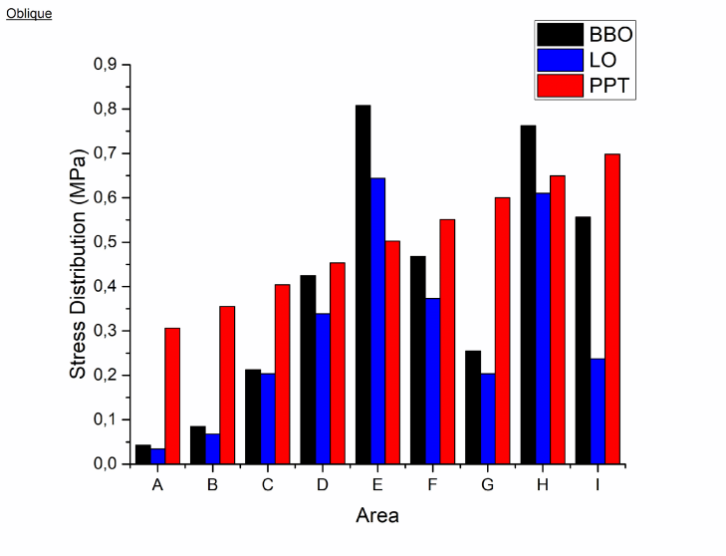
Graph of stress distribution on resorbed alveolar ridge under denture occlusal schemes of bilateral balanced and lingualized when received masticatory load in oblique direction compared to PPT

In the oblique load direction for the BBO occlusal scheme, the resulting stress distribution showed the highest stress concentration in area E of 0.80778 MPa, while for the LO occlusal scheme in area E, it was 0.64394 MPa as seen in Figure 5 and Figure 6. The BBO occlusal scheme showed two areas with stress that exceeded the PPT value, in area E of 0.80778 MPa (PPT 0.50225 MPa) and area H of 0.76256 MPa (PPT 0.64925 MPa). The LO occlusal scheme showed one area with stress that exceeded the PPT value in area E of 0.64394 MPa (PPT 0.50225 MPa). Overall, the BBO occlusal scheme showed a greater stress distribution than the LO occlusal scheme throughout the entire denture supporting area.

## Discussion

Resorption that occurs at the edentulous ridge is a consequence of tooth extraction and the long-term use of ill-fitting dentures ^6^ ^,^ ^22^. A systematic review conducted by Pham et al. ^22^, found that the average resorption in the posterior mandible ranged from 0.01-2.4 mm per year. According to a study by Alsaggaf and Fenlon ^7^, patients who wore dentures for more than five years had significantly more ridge resorption than those who did not. One explanation for this could be that the uneven load distribution exceeded the tolerance level of the underlying mucosa.

Although edentulous patients typically require a masticatory load of roughly 50 N, in some patients this force can reach 80-100 N ^15^ ^,^ ^23^. In their experimental study, Choi et al. ^24^ found that the connective tissue beneath the epithelium would alter first under pressure due to collagen decay, which affects on lowering tissue elasticity and the pain threshold / PPT value. Asking patients to avoid foods that require strong chewing force for mastication is one strategy to help denture users avoid pain, though this cannot always be avoided.

In this study, the stress distribution on the resorbed ridge of the lower jaw under the denture with the BBO occlusal scheme when receiving a load of 100 N in the axial direction showed the largest values in areas A, B, C, E, and H compared to the LO and MO. This was thought to be because more occlusal contact points occur during centric occlusion in the BBO compared to the LO and MO occlusal scheme ^25^, thus the load distributed on the ridge was greater.

The LO showed the largest stress distribution value compared to other occlusal schemes in areas D and F, this was expected because, in this occlusal scheme, the contact point rested on the buccal side of the 1st premolar while in the 2nd premolar and 1st molar, it fell on the central fossa. While the MO showed the largest stress distribution values ​​compared to other occlusal schemes in areas G and I, this was thought to be because of the flat occlusal surfaces, the load pattern was distributed in the anteroposterior direction, which resulted in a higher stress distribution in the posterior area ^26^. Additionally, Madalli et al. ^27^ reported in their in vitro study that the MO occlusal scheme caused more stress on the buccal side, but it still produced a smaller stress distribution when compared to other occlusal schemes.

When associated with PPT, the three occlusal schemes did not show any stress distribution that exceeded the PPT when receiving a load in the axial direction. This was assumed to be because, during centric occlusion, the load was applied vertically and distributed evenly. According to Żmudzki et al. ^15^, the stress distribution produced when receiving a vertical load is still too small to trigger pain. Furthermore, considering that fully edentulous individuals with complete dentures produce masticatory loads ranging between 50-100 N and loads greater than 120 N are not necessary to chew food ^15^ ^,^ ^23^, hence the applied load of 100 N in this study was still within the normal range.

Oblique masticatory loads occur when the individual makes lateral movements, and the direction of this load is directed according to the angle of the maxillary cusps on the mandibular buccal cusp of the posteriors ^19^. BBO occlusal scheme in this study used the anatomical denture teeth with a cusp angle of 33⁰ caused a masticatory load of 100 N to contact in an oblique direction, producing a resultant load of 119 N. This was different from other studies, which used an angle of 45⁰ resulting in a resultant load of 141 N. ^15^. With the same concept, in the LO occlusal scheme, the use of semi-anatomical denture teeth with a cusp angle of 20⁰ caused a resultant load in the oblique direction of 106 N.

The results obtained from this study showed the stress distribution on the resorbed alveolar ridge of the lower jaw under the denture with the BBO occlusal scheme when receiving a resultant load of 119 N in the oblique direction, showing the highest stress concentration on the working side area E (0.80778 MPa) and area H (0.76256 MPa). This value was quite large when compared with previous research by Żmudzki et al. ^15^, which showed a maximum stress distribution of around 0.484 MPa. This was assumed to be because there were several differences with previous studies, such as the condition of the simulated ridge being convex, the thickness of the ridged mucosa being 2 mm, and the determination of the location of the load point only being in the premolar area.

The stress distribution on the resorbed ridge of the lower jaw under the denture with the LO occlusal scheme when receiving a resultant load of 106 N in the oblique direction showed the highest stress concentration on the working side of the premolar area (0.64394 MPa). This value was smaller than the BBO occlusal scheme because the direction of load in the LO occlusion scheme was 20⁰ following the semi-anatomical cusp tilt. According to a study by Mankani et al. ^18^, changes in angulation in the occlusal surface pattern would change the direction of the load and would have an impact on reducing the resultant load; hence, the distribution of stress received by the ridged mucosa would be smaller.

When considered from the perspective of pain, the BBO occlusal scheme produced a stress distribution that exceeded PPT in two areas, namely area E of 0.80778 MPa and area H of 0.76256 MPa. In contrast, in the LO occlusal scheme, there was one area that had a stress distribution that exceeded the PPT, at 0.64394 MPa in area E. According to the randomized clinical trial by Shirani et al. ^28^, patients with this occlusal scheme tend to avoid foods that cause discomfort due to unstable dentures. The instability of the denture with the BBO occlusal scheme is caused by the use of anatomical denture teeth, which results in more resistance occurring during mastication and contributes to uneven distribution of the masticatory load on the ridged mucosa ^12^.

Based on the results of this study, although the MO occlusal scheme showed the lowest stress distribution compared to other occlusal schemes, the LO occlusal scheme only showed one small area that exceeded the PPT, considering that the masticatory load required for mastication is around 50 N on average, and taking into account the matter of aesthetics and masticatory efficiency, the researchers concluded that the LO is the ideal occlusal scheme for fabricating denture with resorbed alveolar ridge conditions.

This study is limited to the three occlusal schemes that are commonly utilized for the teeth arrangement in removable denture fabrication for the resorbed alveolar ridge in the lower jaw and the result was based on the simulation running on finite element method application, which may be needed for the verification even further through the clinical studies.
